# Bioinformatics and system biology approach to identify the influences among COVID-19, ARDS and sepsis

**DOI:** 10.3389/fimmu.2023.1152186

**Published:** 2023-05-16

**Authors:** Peiyu Li, Tao Li, Zhiming Zhang, Xingui Dai, Bin Zeng, Zhen Li, Zhiwang Li

**Affiliations:** ^1^ Department of Gastroenterology, The First People’s Hospital of Chenzhou, Chenzhou, Hunan, China; ^2^ The First Clinical Medical College of Jinan University, Guangzhou, Guangdong, China; ^3^ Department of Critical Care Medicine, The First People’s Hospital of Chenzhou, Chenzhou, Hunan, China; ^4^ Hengyang Medical College, University of South China, Hengyang, Hunan, China; ^5^ Department of Anesthesiology, The First People’s Hospital of Chenzhou, Chenzhou, Hunan, China

**Keywords:** COVID-19, ARDS, sepsis, differentially expressed genes, gene ontology, protein-protein interaction, drug molecule

## Abstract

Background Severe coronavirus disease 2019 (COVID -19) has led to severe pneumonia or acute respiratory distress syndrome (ARDS) worldwide. we have noted that many critically ill patients with COVID-19 present with typical sepsis-related clinical manifestations, including multiple organ dysfunction syndrome, coagulopathy, and septic shock. The molecular mechanisms that underlie COVID-19, ARDS and sepsis are not well understood. The objectives of this study were to analyze potential molecular mechanisms and identify potential drugs for the treatment of COVID-19, ARDS and sepsis using bioinformatics and a systems biology approach. Methods Three RNA-seq datasets (GSE171110, GSE76293 and GSE137342) from Gene Expression Omnibus (GEO) were employed to detect mutual differentially expressed genes (DEGs) for the patients with the COVID-19, ARDS and sepsis for functional enrichment, pathway analysis, and candidate drugs analysis. Results We obtained 110 common DEGs among COVID-19, ARDS and sepsis. ARG1, FCGR1A, MPO, and TLR5 are the most influential hub genes. The infection and immune-related pathways and functions are the main pathways and molecular functions of these three diseases. FOXC1, YY1, GATA2, FOXL, STAT1 and STAT3 are important TFs for COVID-19. mir-335-5p, miR-335-5p and hsa-mir-26a-5p were associated with COVID-19. Finally, the hub genes retrieved from the DSigDB database indicate multiple drug molecules and drug-targets interaction. Conclusion We performed a functional analysis under ontology terms and pathway analysis and found some common associations among COVID-19, ARDS and sepsis. Transcription factors–genes interaction, protein–drug interactions, and DEGs-miRNAs coregulatory network with common DEGs were also identified on the datasets. We believe that the candidate drugs obtained in this study may contribute to the effective treatment of COVID-19.

## Introduction

Coronavirus disease 19 (COVID‐19) is a novel infectious disease caused by severe acute respiratory syndrome coronavirus 2 (SARS‐CoV‐2) (1, 2). The lung is the organ most severely affected by SARS-CoV-2. Patients with COVID-19 autoimmune diseases (3) may develop severe pneumonia or acute respiratory distress syndrome (ARDS). The pathophysiology of those two diseases are characterized by diffuse alveolar damage, exudation, and accompanied by extensive immune cell infiltration and inflammatory cytokine expression (4). If the inflammation is further aggravated, the extrapulmonary organ damage is serious, manifested as multiple organ dysfunction and systemic inflammatory response, its symptoms include cold limbs, microcirculatory dysfunction, weak peripheral pulse, oxidative stress injury, and cytokine storm. This is very similar to sepsis (5). Consideration of severe COVID-19 disease as a sepsis syndrome has relevance and may assist in terms of determining treatments (6).

Sepsis, a systemic inflammatory response syndrome (SIRS) caused by infection, is a common and critical disease with characteristics of high incidence, complex pathogenesis, severe illness, and high mortality (7, 8). In 2016, sepsis3.0 was released (9), which defined sepsis as a clinical syndrome of maladjusted host immune response triggered by infection and manifested as life-threatening organ dysfunction resulting from it. Sepsis is characterized by uncontrolled inflammation and overproduction of reactive oxygen and nitrogen species (RONS), which in turn leads to cell and tissue destruction, immune system dysfunction and pronounced hematopathology, eventually leading to multiple organ failure syndrome (MODS) (10).

Acute respiratory distress syndrome (ARDS) is a serious respiratory disease secondary to trauma, shock, infection and other non-cardiogenic diseases. ARDS is one of the most common and serious complications in the development of sepsis (11). The mortality rate of ARDS is as high as 30%-40% (11). People with COVID-19 who have an autoimmune disease may develop severe pneumonia or ARDS (3).

Given the similarities between COVID-19, ARDS, and sepsis, it is necessary to understand the biological links and underlying molecular mechanisms between the three diseases in order to provide new insights into the pathogenesis of COVID-19 and to search for potential therapeutic agents for patients with COVID-19 or those with COVID-19 secondary to ARDS and sepsis.

**Figure 1:**
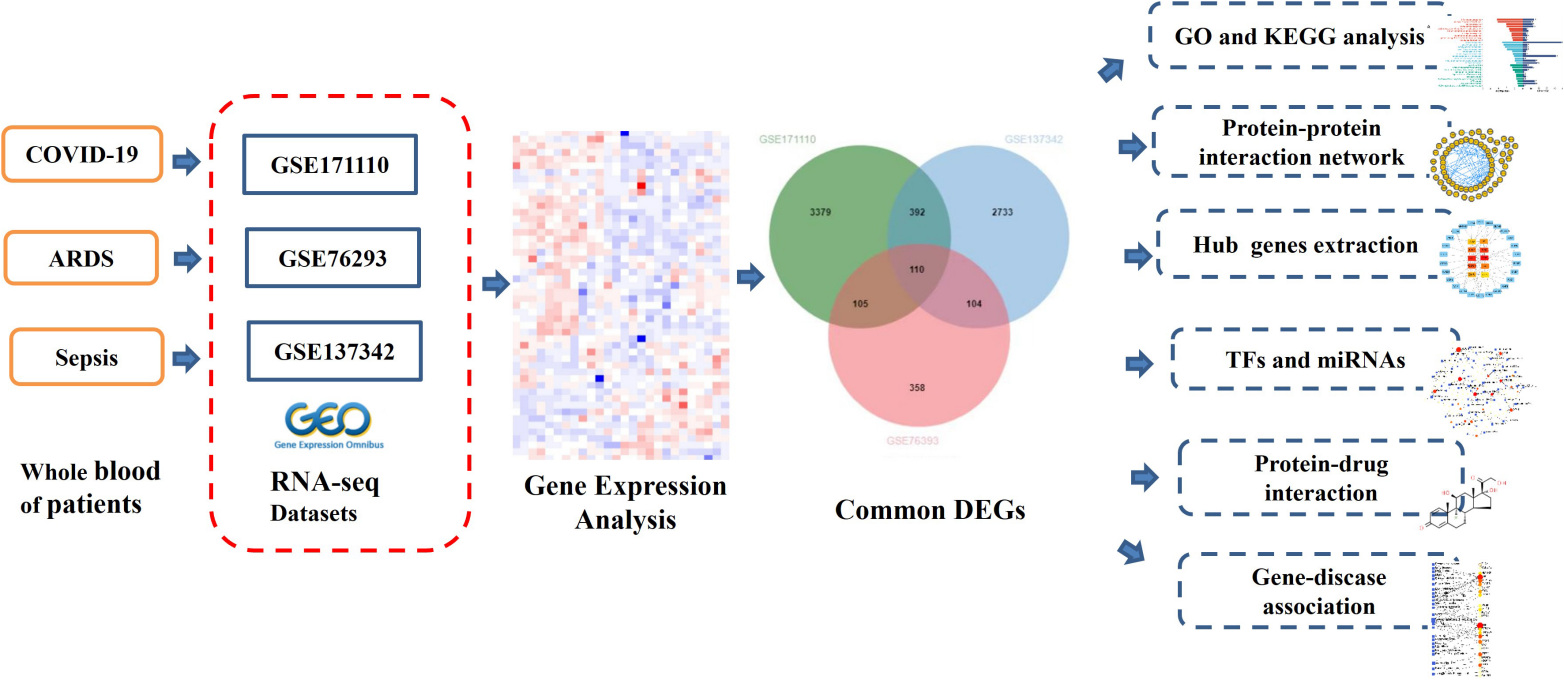
Schematic illustration of the overall general workflow of this study.

In this study, three datasets were used to discover the biological relationship among COVID-19, ARDS and sepsis. The three datasets are GSE171110, GSE76293 and GSE137342. Initially, DEGs were identified for datasets and then found common DEGs genes among the three diseases. The enrichment pathways and biological functions of the common DEGs were analyzed, and the biological processes involved in them were studied. The central gene was extracted from common DEGs, which is an important component of potential drugs. Protein-protein interaction networks (PPIs) are designed by common DEGs to collect central genes. Here, we also trace transcriptional regulators against DEGs similar to GSE171110, GSE76293, and GSE137342. Finally, possible drugs are predicted. The sequential workflow of our research is presented in **Figure 1**.

## Materials and methods

### Collection of the datasets

To analyze shared genetic interrelations and potential therapeutic targets among COVID-19, ARDS and sepsis, we obtained both microarray and RNA-seq datasets from the Gene Expression Omnibus (GEO) database of the National Center for Biotechnology Information (NCBI) (https://www.ncbi.nlm.nih.gov/geo/). The GEO accession ID of the COVID-19 dataset was GSE171110, which included transcriptional profiling from 78 samples (44 COVID-19 samples and 10 healthy control samples, with samples collected from whole blood). GSE171110 was based on the Illumina HiSeq 2500 (Homo sapiens) (GPL16791) platform for extracting RNA sequence analysis. The ARDS dataset was (GEO accession ID: GSE76293) of whole blood containing 12 ARDS patients and 12 healthy controls, which is based on Affymetrix Human Genome U133 Plus 2.0 Array (GPL570) platform. Similarly, the sepsis dataset (GEO accession ID: GSE137342) included array-based gene expression profiles of whole blood from 43 sepsis patients and 12 healthy individuals. **Table 1** shows the basic information of the three datasets.

**Table 1:** Overview of datasets with their geo-features and their quantitative measurements in this analysis.

Disease name	GEO accession	GEO platform	Total DEGs count	Up regulated DEGs count	Down regulated DEGs count
SARS-CoV-2	GSE171110	GPL16791	3986	2620	1366
ARDS	GSE76293	GPL570	677	346	331
Sepsis	GSE137342	GPL10558	3339	3309	30

### Identification of DEGs and common DEGs among COVID-19, ARDS and sepsis

Identification of DEGs for GSE171110, GSE76293 and GSE137342 datasets was the main task of our research. The DEGs for GSE171110 were identified by using the limma package of R programming language. Data generated by microarray analysis were retrieved through DESeq2 and limma package. DEGs for GSE76293 and GSE137342 datasets were analyzed through GEO2R (https://www.ncbi.nlm.nih.gov/geo/geo2r/) web tool which also uses limma package for identifying DEGs. Benjamini–Hochberg false discovery rate (FDR) method was applied to discover genes which were statistically significant and limited false positives. Genes that met the cut-off criteria, adjust P-values <0.01 and |log2FC|≥1.0, were considered as DEGs. Statistical analysis were carried out for each dataset, and the common DEGs of GSE171110, GSE76293 and GSE137342 datasets were obtained using an online VENN analysis tool called Jvenn (http://jvenn.toulouse.inra.fr/app/index.html). Volcano plots were drawn using to show the differential genes in the three datasets.

### Gene ontology and pathway enrichment analysis of DEGs

Gene set enrichment analysis undertakes target gene sets to help understand the general biological functions and chromosomes’ positions. Gene ontology (GO) analysis is a common useful method for functional enrichment analysis (12), which can be classified into biological process (BP), cellular composition (CC) and molecular function (MF). Kyoto Encyclopedia of Genes and Genomes (KEGG) pathway was used for metabolic pathway enrichment analysis and contains considerable utility of genomic analysis (13). GO analysis and KEGG pathway enrichment analysis of DEGs in this study was performed using the DAVID database for annotation, visualization and integrated discovery tools (https://david.ncifcrf.gov/). The adjusted P value < 0.01 was considered statistically significant GO terms and pathways.

### Protein-protein interaction networks and hub genes extraction

The evaluation and analysis of PPI network are fundamental and key to illustrating the molecular mechanisms of key cellular activities. In our study, the PPI networks on common DEGs were identified, and associations between different diseases were found from the perspective of protein interactions. The search tool for the retrieval of interacting genes database called STRING (https://www.string-db.org/) was used to construct the PPI network of proteins derived from shared DEGs among COVID-19, ARDS and sepsis. STRING aims to integrate all known and predicted associations between proteins, including both physical interactions as well as functional associations. This experiment set the medium confidence score of 0.500 to generate the PPI network of common DEGs. The confidence score was also used for the current PPIs network with a medium confidence score of 0.400.

Sebsequently, we consume our PPI network into Cytoscape (v.3.9, https://cytoscape.org/) for a superior visual representation and further PPI network studies. Then, Cytohubba, a plugin in Cytoscape (https://apps.cytoscape.org/apps/cytohubba), was used to calculate the hub genes in the PPI network. Cytohubba can sequence and extract the central or target elements of a biological networks based on different network characteristics. Cytohubba has 11 methods for topological analysis from various viewpoints, and Maximal Clique Centrality (MCC) is the best of them, and the MCC function of Cytohubba was carried out to confirm the top 10 hub genes.

### Identification of transcription factors and miRNAs

Transcription factors (TFs) are proteins that attach to particular genes and control the rate of transcription of genetic information (14). MicroRNAs (miRNAs) are a class of short, endogenously initiated and non-coding RNAs that strive to attach with gene transcripts to affect protein expression; hence, TFs and miRNAs are essential for molecular insights. We used the NetworkAnalyst platform (https://www.networkanalyst.ca/) to construct TF–DEG and DEG–miRNA regulatory networks to analyze relevant TFs and miRNAs. NetworkAnalyst is an extensive online platform for meta-analyzing gene expression data and gaining insights into biological mechanisms, roles, and interpretations. The TF–DEG network was established using the JASPAR database. JASPAR is a publicly available resource for TFs of multiple species in six major taxa. Besides, the DEG–miRNA network was established using the TarBase database. Tarbase and mirTarbase are the main experimental validity databases for miRNAs–target interacting with target genes. We have extracted miRNAs with common DEGs focused on topological analysis from both Tarbase and mirTarbase.

### Drug prediction analysis

Protein-drug interaction (PDI) prediction and drug molecular recognition based on target genes are essential. Potential drug molecules were predicted using the Drug Signatures database (DSigDB) *via* gene set enrichment network tool Enrichr based on the common DEGs of COVID-19, ARDS and sepsis. Enrichr contains a large number of different gene set collections available for analysis, which can be used to explore gene-set enrichment across a genome-wide scale. DSigDB is a web-based resource that contains relevant information about drugs and their target genes for enrichment analysis. This database currently has 22,527 gene sets, including 17,389 drugs and 19,531 genes (15).

### Gene-disease association analysis

DisGeNET is a knowledge management database of gene-disease associations based on various biomedical aspects of diseases, which synchronizes relationships from several origins. It provides and highlights new insights into human genetic disorders. We also examined the gene-disease relationship through NetworkAnalyst using the DisGeNET database to find related diseases and their chronic complications with common DEGs.

## Result

### Identification of DEGs and common DEGs among COVID-19, ARDS and sepsis

**Figure 2:**
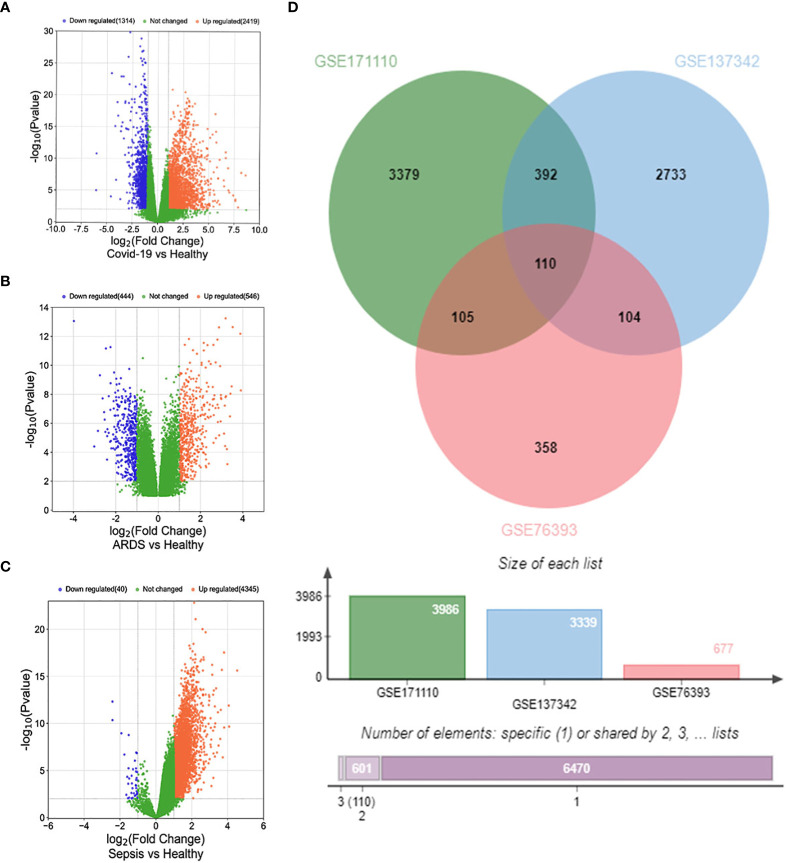
Volcano plots exhibit DEGs of **(A)** COVID-19, **(B)** ARDS and **(C)** sepsis. **(D)** The Venn diagram depicts the common DEGs among COVID-19, ARDS and sepsis.

Firstly, 3986 genes were differentially expressed for COVID-19 from GSE171110, including 2620 up-regulated and 1366 down-regulated genes exposure. In the same way, we identified 677 DEGs (346 up-regulated and 331 down-regulated) in GSE76293 and 3339 DEGs (3309 up-regulated and 30 down-regulated) in GSE137342. The three volcano plots in **Figure 2** visually demonstrated the overall picture of transcribed gene expression for COVID-19, ARDS and sepsis, where red and blue dots indicated up-regulated and down-regulated genes with significant differences, respectively (**Figures 2A–C**). we identified the 110 common DEGs among GSE171110, GSE76293 and GSE137342 (**Figure 2D**). There were some mechanistic commonalities and interaction among COVID-19, ARDS and sepsis, the results of differential expression analysis suggested.

### Gene ontology and pathway enrichment analysis

**Figure 3:**
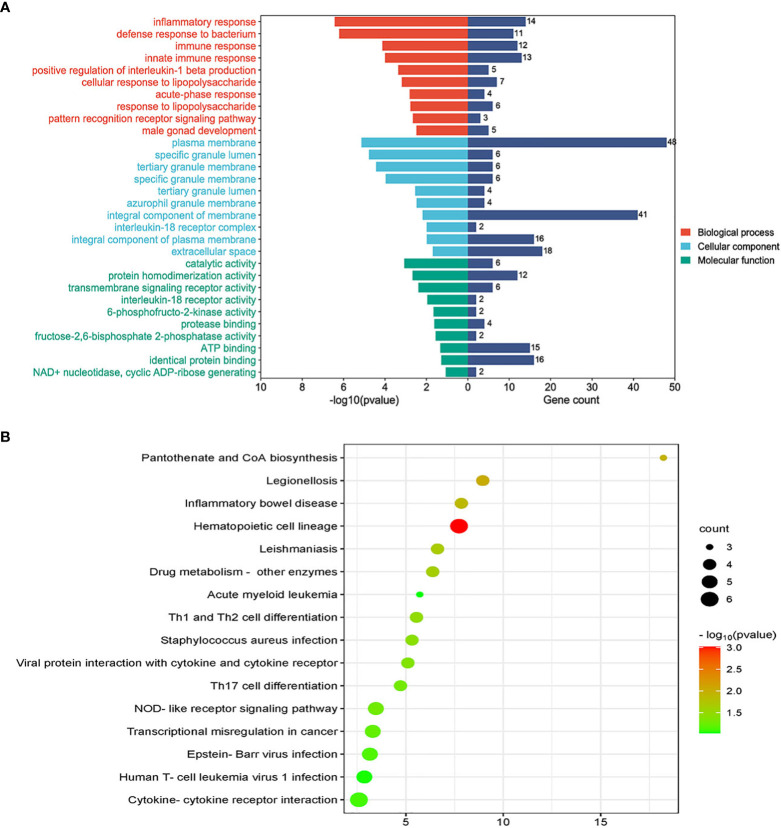
**(A)** The bar graphs of the ontological analysis of the common DEGs among COVID-19, ARDS and sepsis. **(B)** Bubble graphs indicate the results for KEGG analysis based on the common DEGs among COVID-19, ARDS and sepsis.

GO analysis included biological process, cell composition, and molecular function. The GO database was selected as an annotation source. **Table 2** showed the top 10 items in the categories of biological processes, molecular functions, and cell components. **Figure 3A** also showed the top 10 GO terms for molecular function, biological process, and cell compartment, respectively. The differentially expressed genes were significantly enriched in inflammatory response in the subset of BP, enriched in the plasma membrane in the subset of CC, and enriched in catalytic activity in the subset of MF, which were all involved into immunotherapy related functional enrichment.

**Table 2:** Ontological analysis of common DEGs among COVID-19, ARDS and sepsis.

Category	GO ID	Term	P Value	Genes
GO Biological Process	GO:0006954	inflammatory response	3.72E-07	ORM1, SLC11A1, PPBP, NLRC4, LTB4R, TPST1, IL18RAP, AIM2, VNN1, C3AR1, TLR5, IL18R1, CCR3, NAIP
	GO:0042742	defense response to bacterium	6.19E-07	CLEC4D, ANXA3, SLC11A1, HP, LCN2, PPBP, NLRC4, FCGR1A, TLR5, MPO, NAIP
	GO:0006955	immune response	7.58E-05	HLA-DMA, CD4, CLEC4D, IL18RAP, AIM2, SLC11A1, FCGR1A, CST7, CD24, CCR3, IL18R1, LTB4R
	GO:0045087	innate immune response	1.00E-04	ARG1, DEFA4, HMGB2, NLRC4, SRPK1, LILRA5, AIM2, VNN1, LCN2, FCGR1A, TLR5, NAIP, CD177
	GO:0032731	positive regulation of interleukin-1 beta production	4.33E-04	ORM1, AIM2, NLRC4, NAIP, LILRA5
	GO:0071222	cellular response to lipopolysaccharide	6.54E-04	ARG1, DEFA4, HMGB2, LCN2, PPBP, MAPK14, TLR5
	GO:0006953	acute-phase response	0.00153779	CD163, ORM1, HP, LCN2
	GO:0032496	response to lipopolysaccharide	0.001675603	SLC11A1, MGST1, HMGB2, ALPL, IRAK3, MPO
	GO:0002221	pattern recognition receptor signaling pathway	0.002197244	AIM2, NLRC4, NAIP
	GO:0008584	male gonad development	0.003271334	KCNE1, HMGB2, CYP1B1, INSL3, TLR5
GO Cellular Component	GO:0005886	plasma membrane	7.35E-06	KCNE1, FCMR, MGST1, GPR84, ACVR1B, CACNA1E, CD3D, ETS2, LTB4R, LILRA5, ASGR2, MUC1, IL18RAP, PSTPIP2, GRB10, C3AR1, FLVCR2, STOM, CLEC1B, FCGR1A, CCR3, ATP9A, CD177, CD163, CR1, SORT1, ANXA3, SLC11A1, KREMEN1, KCNJ15, AGTRAP, IRAK3, MCEMP1, OLFM4, BMX, SRPK1, F5, CD4, CLEC4D, VNN1, RAB13, TMEM119, SLC26A8, ALPL, RGL4, TLR5, IL18R1, GAS7
	GO:0035580	specific granule lumen	1.70E-05	ORM1, ARG1, DEFA4, HP, LCN2, OLFM4
	GO:0070821	tertiary granule membrane	3.76E-05	CLEC4D, SLC11A1, STOM, MCEMP1, GPR84, CD177
	GO:0035579	specific granule membrane	1.08E-04	CLEC4D, C3AR1, STOM, MCEMP1, GPR84, CD177
	GO:1904724	tertiary granule lumen	0.002875622	ORM1, HP, PPBP, OLFM4
	GO:0035577	azurophil granule membrane	0.003344588	VNN1, MGST1, C3AR1, STOM
	GO:0016021	integral component of membrane	0.006490311	KCNE1, FCMR, MGST1, TMTC1, CD3D, LTB4R, PHTF1, LILRA5, ASGR2, HLA-DMA, MUC1, ZDHHC19, APMAP, C3AR1, CYP1B1, FLVCR2, FCGR1A, SLC37A3, CCR3, ATP9A, CD163, CR1, SORT1, SLC11A1, CSGALNACT2, KREMEN1, KCNJ15, KLHL2, AGTRAP, MCEMP1, FRMD3, CD4, CLEC4D, VNN1, TMEM119, SLC26A8, RGL4, ST6GALNAC3, TLR5, IL18R1, GRINA
	GO:0045092	interleukin-18 receptor complex	0.010283955	IL18RAP, IL18R1
	GO:0005887	integral component of plasma membrane	0.010415288	CD163, CR1, SLC11A1, KCNJ15, GPR84, ACVR1B, LTB4R, MUC1, CD4, C3AR1, SLC26A8, STOM, FCGR1A, CLEC1B, TLR5, CCR3
	GO:0005615	extracellular space	0.020503922	ORM1, CR1, ARG1, DEFA4, HP, HMGB2, PPBP, OLFM4, CST7, MPO, F5, LILRA5, MUC1, LCN2, STOM, ALPL, FAM20A, INSL3
GO Molecular Function	GO:0003824	catalytic activity	8.57E-04	PFKFB2, DDAH2, ECHDC3, UPP1, OLFM4, BCAT1
	GO:0042803	protein homodimerization activity	0.002126894	TPST1, CD4, TP53I3, GADD45A, DEFA4, SLC11A1, STOM, IRAK3, NLRC4, CST7, GYG1, UPB1
	GO:0004888	transmembrane signaling receptor activity	0.004116847	CD4, FCMR, FCGR1A, CLEC1B, TLR5, CD3D
	GO:0042008	interleukin-18 receptor activity	0.011098693	IL18RAP, IL18R1
	GO:0003873	6-phosphofructo-2-kinase activity	0.022075366	PFKFB2, PFKFB3
	GO:0002020	protease binding	0.024084899	LCN2, INSL3, ATP9A, CD177
	GO:0004331	fructose-2,6-bisphosphate 2-phosphatase activity	0.02751836	PFKFB2, PFKFB3
	GO:0005524	ATP binding	0.047221652	PFKFB2, PFKFB3, TDRD9, IRAK3, NLRC4, BMX, MAPK14, OPLAH, ACVR1B, SRPK1, PGS1, MKNK1, KIF1B, NAIP, ATP9A
	GO:0042802	identical protein binding	0.052207838	ARG1, MGST1, KLHL2, AGTRAP, MCEMP1, NLRC4, OPLAH, CD3D, CD4, AIM2, GRB10, LCN2, STOM, UPP1, BCAT1, GAS7
	GO:0061809	NAD+ nucleotidase, cyclic ADP-ribose generating	0.085446381	IL18RAP, IL18R1

Top 10 terms of each category are listed.

KEGG pathway analysis revealed the following top 10 pathways: Hematopoietic cell lineage, Legionellosis, Pantothenate and CoA biosynthesis, Inflammatory bowel disease, Leishmaniasis, Drug metabolism-other enzymes, Th1 and Th2 cell differentiation, Staphylococcus aureus infection, Viral protein interaction with cytokine and cytokine receptor and Th17 cell differentiation. **Table 3** listed the KEGG enrichment pathways generated from the selected dataset. For a more detailed illustration, the pathway enrichment analysis was showed in the bubble graphs (**Figure 3B**).

**Table 3:** Pathway enrichment analysis of common DEGs among COVID-19, ARDS and sepsis.

Category	Pathways	P Value	Genes
KEGG_PATHWAY	Hematopoietic cell lineage	9.55E-04	HLA-DMA, CD4, CR1, FCGR1A, CD24, CD3D
	Legionellosis	0.009529899	CR1, NLRC4, TLR5, NAIP
	Pantothenate and CoA biosynthesis	0.011199108	VNN1, BCAT1, UPB1
	Inflammatory bowel disease	0.013620921	HLA-DMA, IL18RAP, TLR5, IL18R1
	Leishmaniasis	0.021374347	HLA-DMA, CR1, FCGR1A, MAPK14
	Drug metabolism - other enzymes	0.023620807	MGST1, UPP1, MPO, UPB1
	Th1 and Th2 cell differentiation	0.033841345	HLA-DMA, CD4, MAPK14, CD3D
	Staphylococcus aureus infection	0.037683794	HLA-DMA, DEFA4, C3AR1, FCGR1A
	Viral protein interaction with cytokine and cytokine receptor	0.041740915	IL18RAP, PPBP, CCR3, IL18R1
	Th17 cell differentiation	0.050488231	HLA-DMA, CD4, MAPK14, CD3D
	NOD-like receptor signaling pathway	0.053210438	AIM2, DEFA4, NLRC4, MAPK14, NAIP
	Transcriptional misregulation in cancer	0.061334532	CCNA1, GADD45A, DEFA4, FCGR1A, MPO
	Epstein-Barr virus infection	0.070083846	CCNA1, HLA-DMA, GADD45A, MAPK14, CD3D
	Cytokine-cytokine receptor interaction	0.076591926	CD4, IL18RAP, PPBP, ACVR1B, CCR3, IL18R1
	Human T-cell leukemia virus 1 infection	0.091699112	CCNA1, HLA-DMA, CD4, CD3D, ETS2
	Acute myeloid leukemia	0.094203186	CCNA1, FCGR1A, MPO

### Classification of hub proteins and submodule

**Figure 4:**
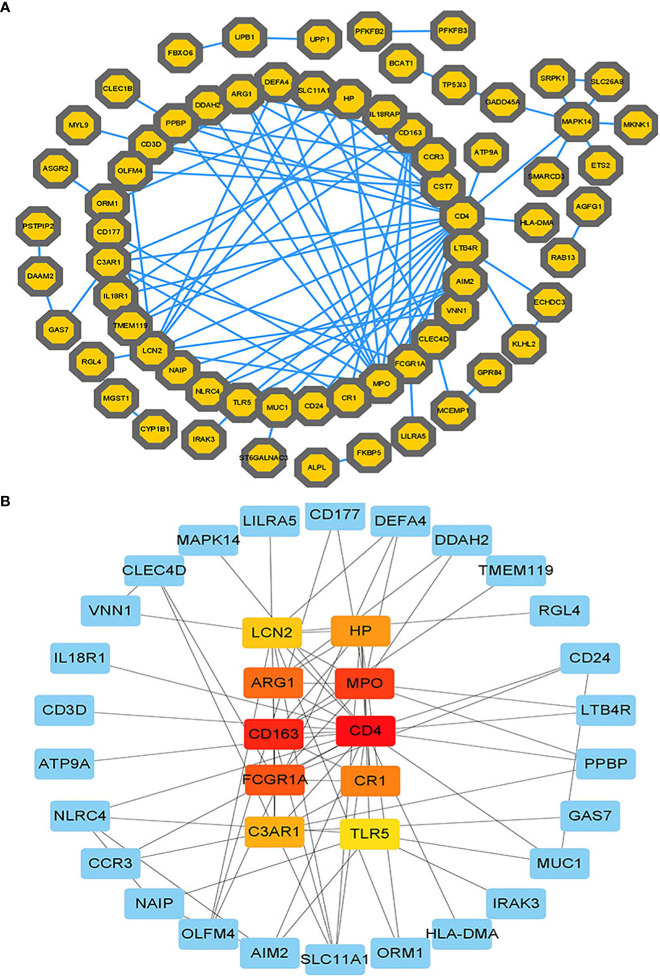
**(A)** The PPI network of common DEGs among COVID-19, ARDS and sepsis. In the figure, the octagonal nodes represent DEGs and edges represent the interactions between nodes. The PPI network was generated using String and visualized in Cytoscape. **(B)** The hub genes were identified from the PPI network using the Cytohubba plug in Cytoscape. Here, the colored nodes represent the highlighted top 10 hub genes and their interactions with other molecules.

The PPI network of common DeGs included 110 nodes and 105 edges, as shown in **Figure 4A**. Based on PPI network analysis, we identified the top 10 DEGs as the most influential genes by using the Cytohubba plugin in Cytoscape. The hub genes were namely LCN2, HP, ARG1, MPO, CD163, CD4, FCGR1A, CR1, C3AR1, and TLR5. These hub genes could serve as potential biomarkers and potentially new therapeutic strategies for studying disease. The expanded network of hub – gene interactions derived from the PPI network was shown in **Figure 4B**.

### Construction of regulatory networks

**Figure 5:**
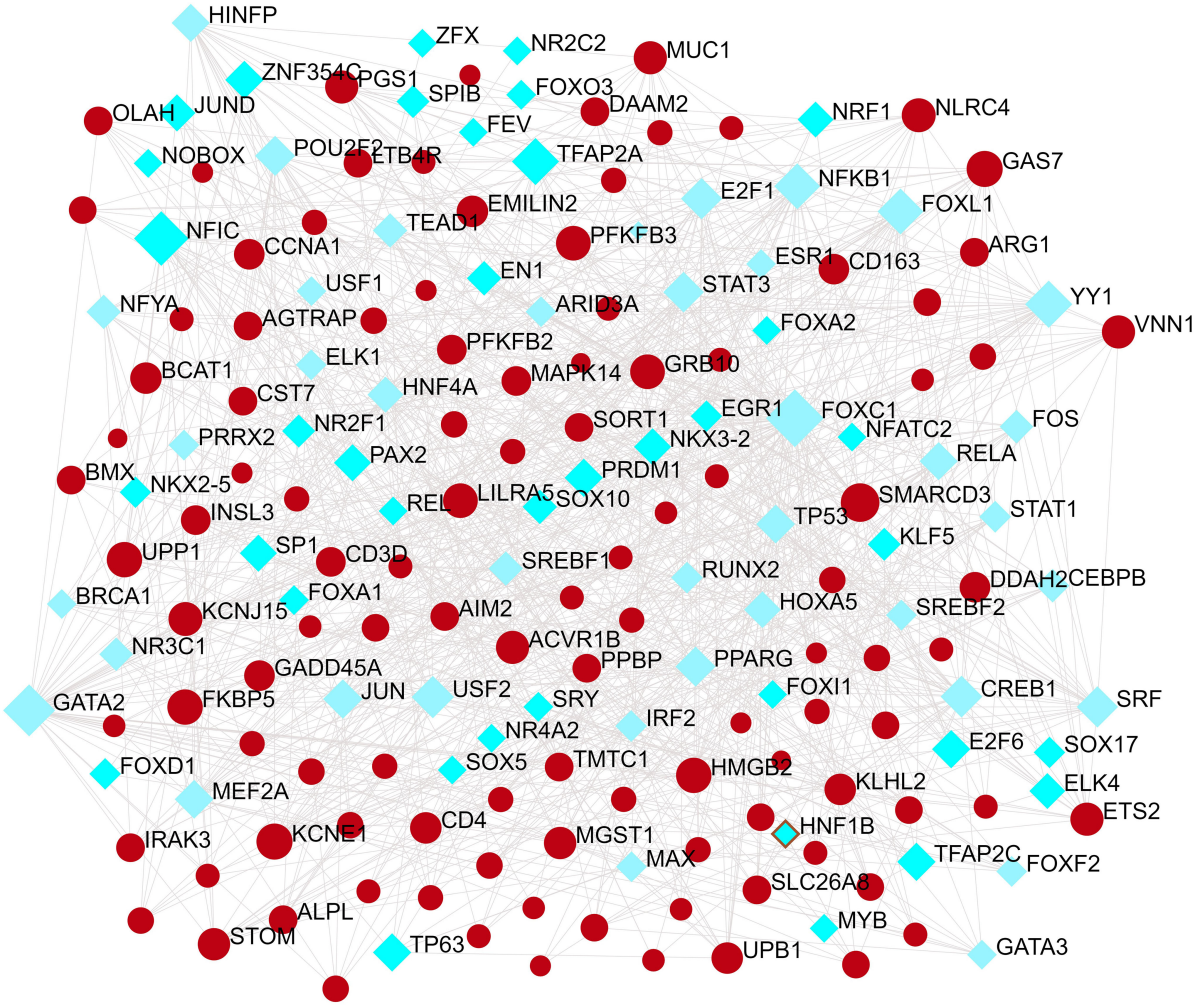
The Network Analyst created an interconnected regulatory interaction network of DEG-TFs. In it, blue square nodes represent TFs, gene symbols interact with TFs as yellow circle nodes.

TFs regulators interaction with the common DEGs was pictured in **Figure 5**. From the **Figure 5**, KCNJ15, SMARCD3, LILRA5, GAS7 and HMGB2 were more abundant in the highly expressed DEGs as these genes have a higher degree in the TF–gene interactions network. TFs such as FOXC1, GATA2, YY1, FOXL1, FOXO3, STAT1 and STAT3 were more significant than others as presented in the same **Figure 5**.

**Figure 6:**
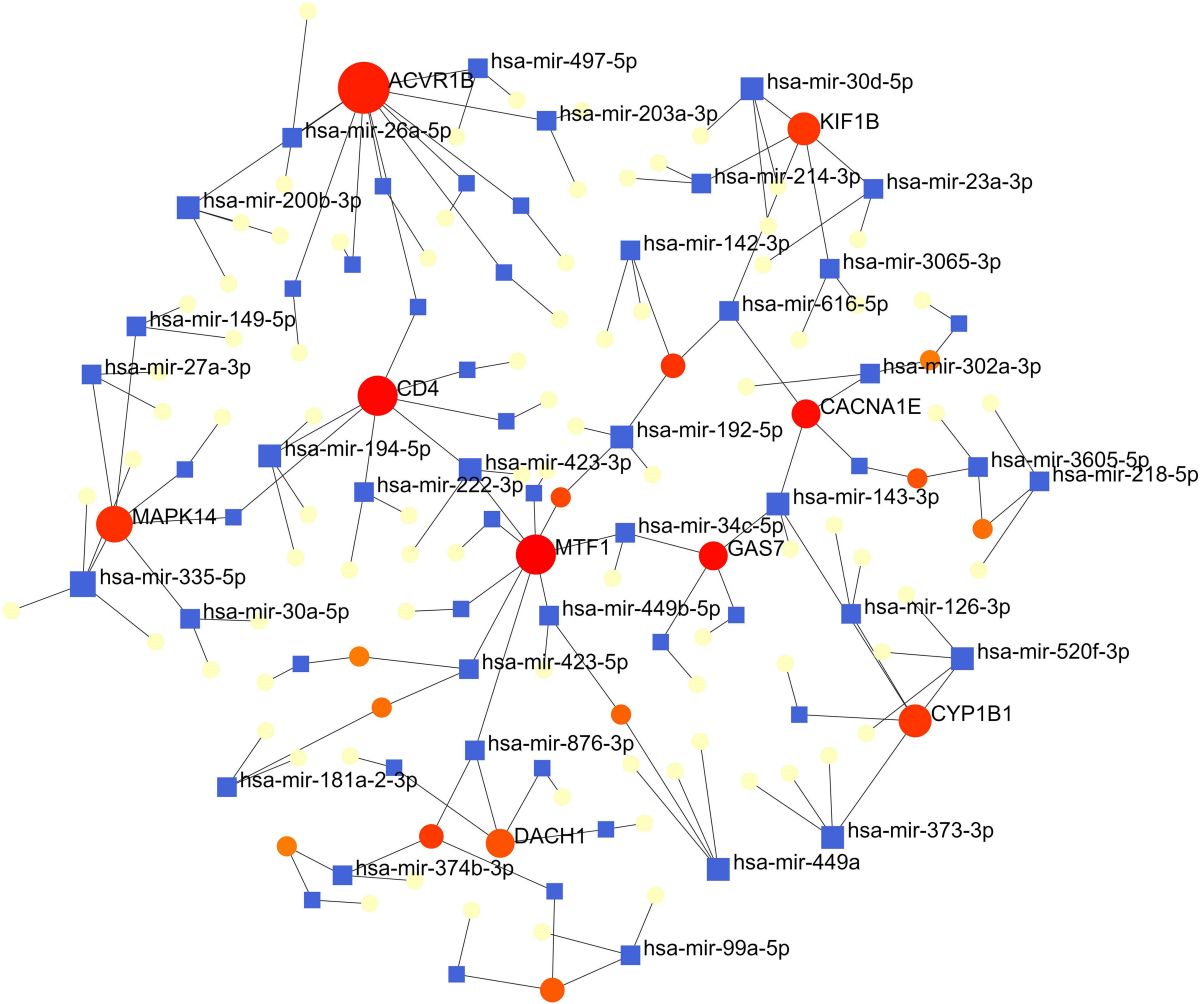
The interconnected regulatory interaction network of DEGs-miRNAs. blue squares represented miRNA s, while red circles represented DEGs.

The interactions of miRNAs regulators with common DEGs was showed in the **Figure 6**. In the **Figure 6**, blue squares represented miRNAs and red circles represented DEGs. Our results showed that ACVR1B, MTF1, CD4, MAPK14, DACH1, KIF1B, GAS7 and CYP1B1 were the hub genes of this network, with the five genes most involved in miRNAs. Besides, we also detected the significant hub miRNAs from the miRNAs-gene interaction network, namely mir-335-5p, hsa-mir-26a-5p, hsa-mir-200b-3p, hsa-mir-194-5p, hsa-mir-192-5p, hsa-mir-143-3p and hsa-mir-520f-3p.

### Identification of candidate drugs

Based on transcriptome signatures, we identified 10 possible drug molecules using Enrichr from the DSigDB database. The top 10 chemical compounds were extracted based on their P-value. These 10 possible drug molecules included isoflupredone, etynodiol, fludroxycortide, flunisolide, halcinonide, flumetasone, diflorasone, ribavirin, gabexate and alclometasone (**Table 4**).

**Table 4:** List of the suggested drugs for COVID-19.

Term	P-value	Chemical Formula	Structure
isoflupredone HL60 UP	2.76E-11	C21H27FO5	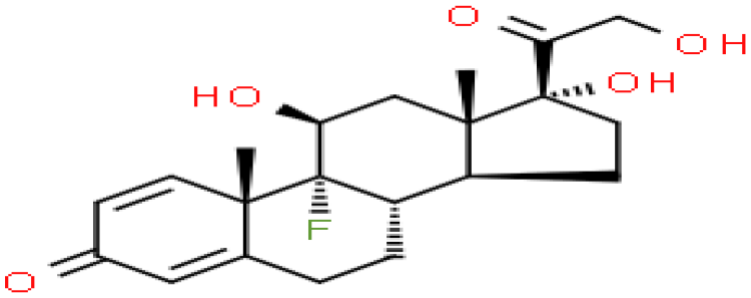
etynodiol HL60 UP	6.10E-11	C20H28O2	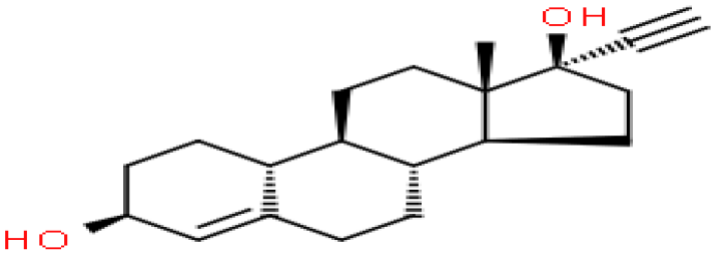
fludroxycortide HL60 UP	1.35E-10	C24H33FO6	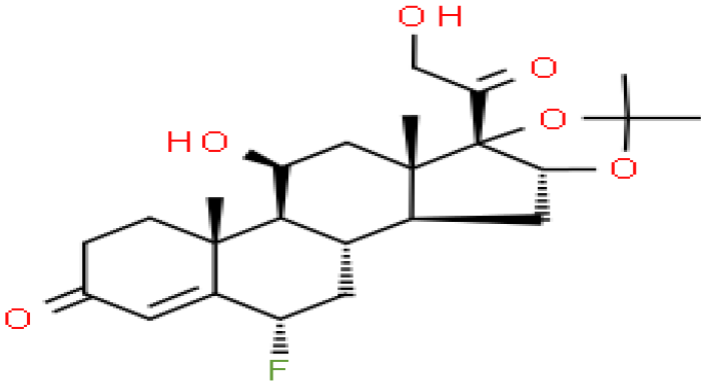
flunisolide HL60 UP	5.08E-09	C24H31FO6	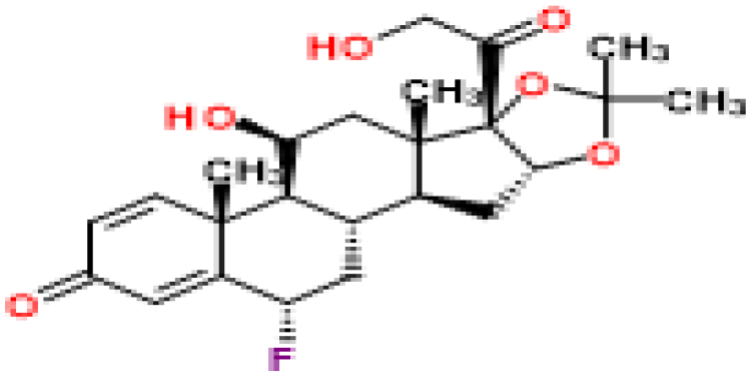
halcinonide HL60 UP	6.50E-09	C24H32ClFO5	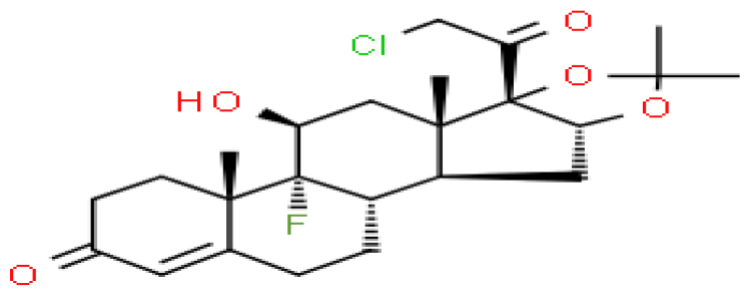
flumetasone HL60 UP	8.23E-09	C22H28F2O5	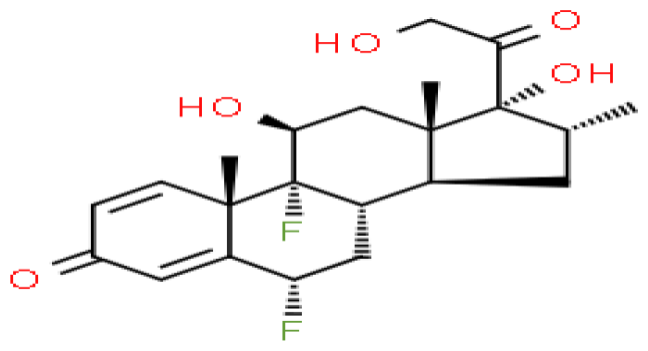
diflorasone HL60 UP	1.03E-08	C26H32F2O7	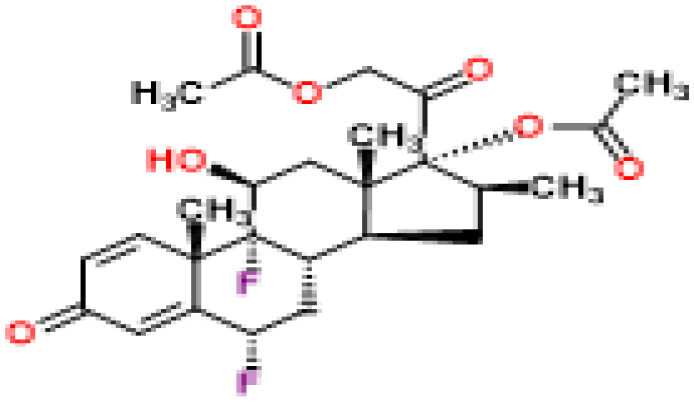
ribavirin HL60 UP	1.03E-08	C8H12N4O5	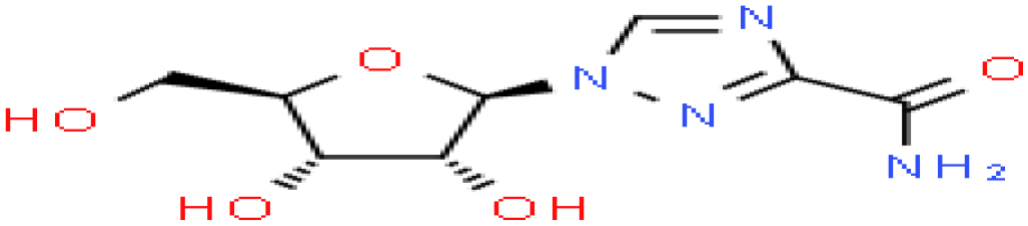
gabexate HL60 UP	1.29E-08	C16H23N3O4	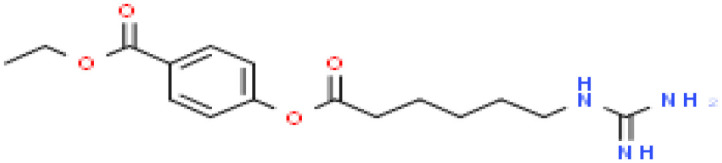
alclometasone HL60 UP	2.86E-08	C22H29ClO5	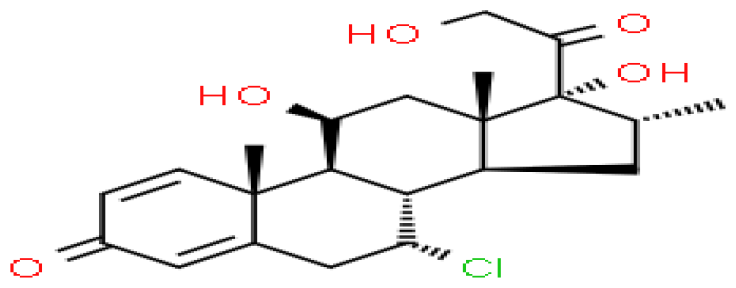

### Identification of disease association

**Figure 7:**
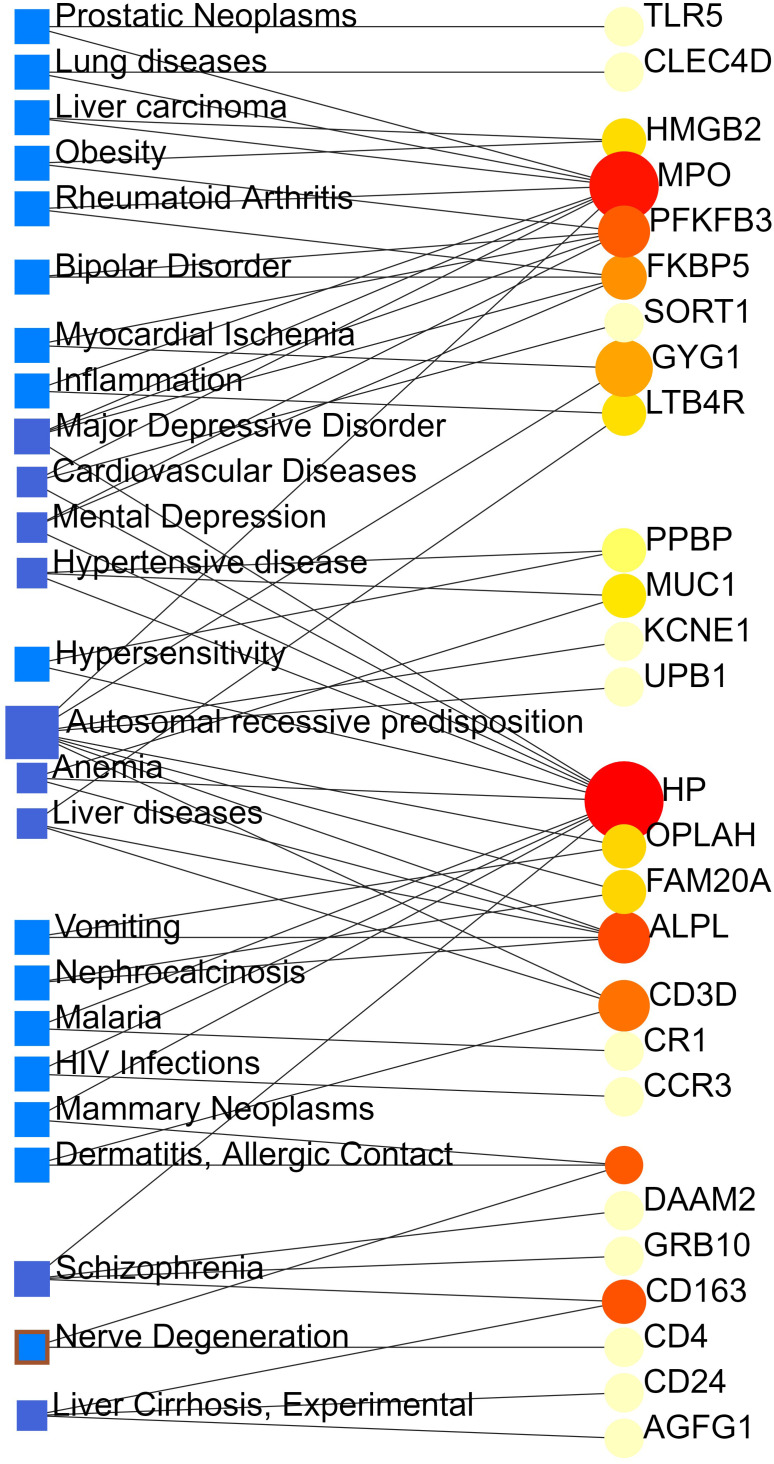
The gene-disease association network represents diseases associated with common DEGs. The disorders depicted by the square node and also its subsequent gene symbols are defined by the circular node.

From the analysis of the gene-disease association by Network Analyst (16), we noticed that major depressive disorder, cardiovascular diseases, mental depression, hypertensive disease, autosomal recessive predisposition, anemia, liver diseases, schizophrenia and liver cirrhosis are most coordinated to our reported hub genes, and even among COVID-19, ARDS and sepsis. The gene-disease association was shown in **Figure 7**.

## Discussion

Most patients with severely ill COVID-19 eventually develop typical septic shock manifestations, including cold limbs, microcirculatory dysfunction, peripheral pulse weak, oxidative stress injury, and cytokine storm (17). These symptoms and serological markers are present in both ARDS and sepsis patients (18). ARDS induced by COVID-19 can progress to sepsis (17).

The results of our GO analysis from the DAVID show that inflammatory response (14 genes), defense response to bacterium (11 genes), immune response (12 genes) and innate immune response (13 genes) are among the top GO terms. Innate immune cell hyperactivation plays a critical role in the pathogenesis of severe COVID-19 (19). Studies have shown that the infection mediated immuno-compromised state can result in poor clinical morbidity and a high risk of fatal pneumonia (20). In the molecular function experiment, catalytic activity, protein homodimerization activity and transmembrane signaling receptor activity are three top GO pathways. According to the cellular component, top GO terms are plasma membrane, specific granule lumen, tertiary granule membrane and specific granule membrane.

By identifying the KEGG pathways for 110 common DEGs, similar pathways were identified for COVID-19, ARDS, and sepsis. Some patients experiencing severe COVID-19, the disease caused by the SARS-CoV-2 beta coronavirus, develop what is sometimes described as a “cytokine storm” or “cytokine release syndrome” (21). These cytokines produce eosinopenia and lymphocytopenia characterized by low counts of eosinophils, CD8+ T cells, natural killer (NK) and naïve T-helper cells, simultaneously inducing naive B-cell activation, increased T-helper cell 17 (Th17) lymphocyte differentiation and the stimulation of monocyte and neutrophil recruitment (18, 22).

The top hub proteins indicate different diseases, most risk factors for the COVID-19, ARDS and sepsis. A total of 10 hub-proteins (LCN2, HP, ARG1, MPO, CD163, CD4, FCGR1A, CR1, C3AR1 and TLR5) identified involved in these diseases. ARG1 can be released to the extracellular microenvironment during inflammatory conditions, e.g., asthma and infectious diseases (23). FCGRIA has been proposed as an attractive target for immunotherapy by various workers (24). Research shows that infiltrating neutrophils, a hallmark of COVID-19, can release myeloperoxidase (MPO), which can activate several pathways that lead to elevated cytokines and production of ROS such as hypochlorous acid (HOCl), superoxide (O2•-), and hydrogen peroxide (H2O2) (25). Another possible facet of the observed pathophysiology in critical cases of COVID-19 is a decline in nitric oxide (NO) and combined with the effect of excessive ROS on the structure and function of hemoglobin (Hb) could impact pulmonary and peripheral circulation, possibly eventually leading to critical or fatal hypoxia (26). Chakraborty recommend the use of active immunomodulation through TLR5 and activation of the innate immune to fight against SARS‐CoV‐2 as the main entry point of this virus is angiotensin‐converting enzyme 2 receptor respiratory in epithelial cells (27).

To understand how common DEGs regulate COVID-19 (or ARDS, sepsis) at the transcriptional level, the interactions among TFs, miRNAs and genes were investigated *via* web tools. The identified TFs, such as FOXC1, GATA2, YY1, FOXL1, FOXO3, STAT1 and STAT3, are associated with COVID-19. In previous bioinformatics analysis, Ahmed (28) and Islam et al. (29) both found that FOXC1, YY1, GATA2, and FOXL1 are important TFs for COVID-19. Coincidentally, Lu Lu also found that FOXC1, YY1, GATA2 and FOXL1 are important TFs for COVID-19 (30). After a careful review of the scientific literature, we realized that COVID-19 is a disease caused by a catastrophic cascade of failures stemming from the SARS-CoV-2- mediated dysregulation of STATs. Specifically, the dysfunctions of STAT1 and STAT3 induced by SARS-CoV-2 proteins may be the foundation of severe COVID-19 pathophysiology (31).

Our results also showed that the regulatory relationship between miRNAs (mir-335-5p, hsa-mir-26a-5p, hsa-mir-200b-3p, hsa-mir-194-5p, hsa-mir-192-5p, hsa-mir-143-3p and hsa-mir-520f-3p) and genes (ACVR1B, MTF1, CD4, MAPK14, DACH1, KIF1B, GAS7 and CYP1B1) that may play important roles in COVID-19, ARDS and sepsis. It was worth noting that Huan Hu et al. predicted that mir-335-5p associated with different genes from COVID-19 (7). Laura Teodori et al. showed through bioinformatics analysis that miR-335-5p are regulated by Spike, ACE and histone deacetylation (HDAC) pathway (32). Upregulation of hsa-mir-26a-5p expression was significantly associated with inflammatory responses and cytokine - and chemokine-mediated signaling pathways in the sera of lactating mothers with type 1 diabetes (33).

We performed gene-disease (GD) analyses and predicted significant DEGs associations with different diseases. Diseases enriched by these DEGs include: major depressive disorder, mental depression, schizophrenia, cardiovascular diseases, hypertensive disease, anemia, liver diseases and liver cirrhosis. Recent studies have proven that people with mental illness, especially depression and schizophrenia, are at high risk of being infected by COVID-19 (34). According to the Clinical Bulletin of the American College of Cardiology (ACC), the mortality rate for patients with coexisting hypertension or cardiovascular disease COVID-19 was 6.0% and 10.5%, respectively (35). Besides, 16.7% of patients face arrhythmia, and 7.2% developed acute cardiac problems with COVID 19-associated complications (36). A study reported that 2–11% of COVID-19 patients had primary chronic liver disease (37). Of those diagnosed with COVID-19, about one-third of cirrhosis patients die within 10 days, and two-thirds of cirrhosis patients died before admission to the intensive care unit (38).

The current crisis of the COVID-19 pandemic around the world has been devastating as many lives have been lost to the novel SARS CoV-2 virus. Thus, There are bioinformatics studies that aim to identify promising treatment options for COVID-19 through computational drug reuse. Alfred Olaoluwa Akinlalu’s study predicted that ethynodiol diacetate exhibited better binding energy and pharmacokinetic properties than the off-Wlabel reference drugs (hydroxychloroquine, lopinavir and remdesivir) which has been currently investigated for the treatment of COVID-19 (39). Giuseppe Nunnari has highlighted Flunisolide, Thalidomide, Lenalidomide, Desoximetasone, xylazine, and salmeterol as potential drugs against SARS-CoV (40). Seyedeh Zahra Mousavi’s research showed that HDAC inhibitors can be an effective drug against COVID-19 (41). The mechanism of action needs further investigation.

## Conclusions

We performed a functional analysis under ontology terms and pathway analysis and found some common associations among COVID-19, ARDS and sepsis. Transcription factors–genes interaction, protein–drug interactions, and DEGs-miRNAs coregulatory network with common DEGs also identified on the datasets. We believe that the candidate drugs obtained in this study may contribute to the effective treatment of COVID-19. So, our identified genes can be a novel therapeutic target for COVID-19 vaccine development.

## Data availability statement

Publicly available datasets were analyzed in this study. The data could be downloaded from the GEO database of the National Center for Biotechnology Information (NCBI) (https://www.ncbi.nlm.nih.gov/geo/), accession numbers GSE171110, GSE76293, and GSE137342.

## Author contributions

ZhiL conceived and designed the study. PL and TL provided equal contributions to research design, data analysis, and article writing. ZZ and XD helped to write the manuscript. All authors contributed to the article and approved the submitted version.
