# Corrigendum: Bioinformatics and system biology approach to identify the influences among COVID-19, ARDS and sepsis

**DOI:** 10.3389/fimmu.2023.1236992

**Published:** 2023-06-20

**Authors:** Peiyu Li, Tao Li, Zhiming Zhang, Xingui Dai, Bin Zeng, Zhen Li, Zhiwang Li

**Affiliations:** ^1^ Department of Gastroenterology, The First People’s Hospital of Chenzhou, Chenzhou, Hunan, China; ^2^ The First Clinical Medical College of Jinan University, Guangzhou, Guangdong, China; ^3^ Department of Critical Care Medicine, The First People’s Hospital of Chenzhou, Chenzhou, Hunan, China; ^4^ Hengyang Medical College, University of South China, Hengyang, Hunan, China; ^5^ Department of Anesthesiology, The First People’s Hospital of Chenzhou, Chenzhou, Hunan, China

**Keywords:** COVID-19, ARDS, sepsis, differentially expressed genes, gene ontology, protein-protein interaction, drug molecule

In the published article, there was an error in affiliation 1. Instead of “Department of Gastroenterology, The First Peoples’s Hospital of Chenzhou, Chenzhou, Hunan, China.”, it should be “Department of Gastroenterology, The First People’s Hospital of Chenzhou, Chenzhou, Hunan, China.”.

In the published article, there was an error in affiliation 3. Instead of “Department of Critical Care Medicine, The First Peoples’s Hospital of Chenzhou, Chenzhou, Hunan, China.”, it should be “Department of Critical Care Medicine, The First People’s Hospital of Chenzhou, Chenzhou, Hunan, China.”.

In the published article, there was an error in affiliation 5. Instead of “Department of Anesthesiology, The First Peoples’s Hospital of Chenzhou, Chenzhou, Hunan, China.”, it should be “Department of Anesthesiology, The First People’s Hospital of Chenzhou, Chenzhou, Hunan, China.”.

In the published article, there was an error in the Funding statement. Instead of “National Science Foundation of Hunan Province” it should be “Natural Science Foundation of Hunan Province”. The correct Funding statement appears below.

